# Treat-to-target urate-lowering therapy and hospitalizations for gout: results from a nationwide cohort study in England

**DOI:** 10.1093/rheumatology/keac638

**Published:** 2022-11-10

**Authors:** Mark D Russell, Edward Roddy, Andrew I Rutherford, Benjamin Ellis, Sam Norton, Abdel Douiri, Martin C Gulliford, Andrew P Cope, James B Galloway

**Affiliations:** Centre for Rheumatic Diseases, King’s College London, London, UK; School of Medicine, Keele University, Keele, UK; Department of Rheumatology, King’s College Hospital NHS Foundation Trust, London, UK; Department of Rheumatology, Imperial College Healthcare NHS Foundation Trust, London, UK; Centre for Rheumatic Diseases, King’s College London, London, UK; School of Population Health and Environmental Sciences, King’s College London, London, UK; School of Population Health and Environmental Sciences, King’s College London, London, UK; Centre for Rheumatic Diseases, King’s College London, London, UK; Centre for Rheumatic Diseases, King’s College London, London, UK

**Keywords:** gout, crystal arthritis, hospitalizations, urate-lowering therapy, treat-to-target

## Abstract

**Objective:**

To investigate associations between treat-to-target urate-lowering therapy (ULT) and hospitalizations for gout.

**Methods:**

Using linked Clinical Practice Research Datalink and NHS Digital Hospital Episode Statistics data, we described the incidence and timing of hospitalizations for flares in people with index gout diagnoses in England from 2004–2020. Using Cox proportional hazards and propensity models, we investigated associations between ULT initiation, serum urate target attainment, colchicine prophylaxis, and the risk of hospitalizations for gout.

**Results:**

Of 292 270 people with incident gout, 7719 (2.64%) had one or more hospitalizations for gout, with an incidence rate of 4.64 hospitalizations per 1000 person-years (95% CI 4.54, 4.73). There was an associated increased risk of hospitalizations within the first 6 months after ULT initiation, when compared with people who did not initiate ULT [adjusted Hazard Ratio (aHR) 4.54; 95% CI 3.70, 5.58; *P* < 0.001]. Hospitalizations did not differ significantly between people prescribed *vs* not prescribed colchicine prophylaxis in fully adjusted models. From 12 months after initiation, ULT associated with a reduced risk of hospitalizations (aHR 0.77; 95% CI 0.71, 0.83; *P* < 0.001). In ULT initiators, attainment of a serum urate <360 micromol/l within 12 months of initiation associated with a reduced risk of hospitalizations (aHR 0.57; 95% CI 0.49, 0.67; *P* < 0.001) when compared with people initiating ULT but not attaining this target.

**Conclusion:**

ULT associates with an increased risk of hospitalizations within the first 6 months of initiation but reduces hospitalizations in the long term, particularly when serum urate targets are achieved.

Rheumatology key messages

- Urate-lowering therapy reduces hospitalizations for gout flares in the long term, particularly when urate targets are achieved.
- Only a minority of patients attain serum urate targets within a year of discharge.
- Strategies are needed to encourage the uptake of treat-to-target urate-lowering therapy.

## Introduction

Hospitalizations for gout flares have increased markedly in recent years, on a background of an increasing prevalence of gout and sub-optimal management [1–3]. In England, hospitalizations for gout doubled between 2006 and 2020 [4]. Hospitalizations due to gout also doubled in the United States, Canada and Sweden, contrasting large decreases in admissions for rheumatoid arthritis [5–7]. Despite this, few studies have investigated strategies to prevent avoidable gout admissions [8].

Gout is unique among the inflammatory arthritides, in that there are curative medications that prevent flares: urate-lowering therapies (ULT), such as allopurinol and febuxostat. The benefits of ULT are well recognized in primary care settings. A large, randomized controlled trial (RCT) demonstrated that ULT, when titrated to achieve a serum urate (SU) below the saturation threshold for crystal formation (<360 micromol/l), significantly reduced the frequency of gout flares at 2 years compared with usual care [risk ratio (RR) 0.33; 95% CI 0.19, 0.57] [9]. However, in the short term, initiation and titration of ULT can precipitate flares: the frequency of gout flares with treat-to-target ULT in the first year of this trial exceeded that observed with usual care (RR 1.36; 95% CI 1.05, 1.77).

What is not known is whether treat-to-target ULT prevents hospitalizations for gout. Using a population-level dataset with over 290 000 people with incident gout, we investigated two primary objectives: (i) the impact of ULT, with and without colchicine prophylaxis, on the risk of hospitalizations for gout; and (ii) whether attaining target SU levels influences the risk of hospitalizations following ULT initiation.

## Patients and methods

### Data source

The Clinical Practice Research Datalink (CPRD) is a longitudinal health database with pseudonymised demographic, clinical and prescription data from people registered with over 2000 UK primary care practices [10]. We used CPRD Aurum, containing data on 41 million people currently or previously registered with general practices that use EMIS Web^®^ health record software. Currently registered patients (13.3 million) in CPRD Aurum cover 20% of the UK population, with 99% of contributing practices registered in England [11].

Primary care data in CPRD Aurum was linked to National Health Service (NHS) Digital Hospital Episode Statistics Admitted Patient Care (HES APC) data. HES APC contains pseudonymised data on all admissions and attendances at English NHS providers, including acute hospital trusts.

### Study population and case definition

We conducted a population-level, observational cohort study of people aged ≥18 years, currently or previously registered with a CPRD Aurum practice, who had index gout diagnoses between 1 January 2004 and 31 December 2020, and who were eligible for linkage to secondary care data. The start date of 2004 corresponds to the more widespread availability of laboratory-linked data with the incorporation of the Quality and Outcomes Framework into UK primary care contracts. Linked secondary care data was available to 31 March 2021, with 98% of patients being eligible for linkage.

We defined an index gout diagnosis as a new diagnostic code for gout in people without previous gout diagnostic codes (see Supplementary Data S1, available at *Rheumatology* online, for codelists). At least 12 months of registration with a CPRD Aurum practice prior to the first gout code was required, to ensure only incident cases were detected, in addition to ≥12 months of follow-up post-diagnosis.

### Definition of hospitalizations

We defined a hospitalization for gout flare as an admission episode with a primary gout diagnosis [International Classification of Diseases version 10 (ICD10) code: M10]. We did not include admissions with only secondary diagnoses of gout or emergency department (ED)-only attendances, due to less reliable coding [12]. We excluded admissions that occurred within 7 days of another gout admission, to reduce capture of re-admissions for single flares. Only admission episodes within patients’ CPRD registration windows were included. People newly diagnosed with gout during a hospitalization will typically have a gout code entered in primary care following receipt of the discharge notification; in these cases, we selected the admission date as the index diagnosis date.

We classified gout hospitalizations into: (i) index diagnosis events: i.e., first recorded diagnosis of gout made during or within 7 days of a hospitalization for flare; and (ii) non-index events: hospitalizations ≥7 days after the initial diagnosis.

### Incidence rate of hospitalizations

We reported the proportion of patients who had one or more hospitalizations for gout, and the number of hospitalizations during the study period. We described patients’ characteristics at diagnosis (without inferential statistics) for the whole study cohort and, separately, for patients who had one or more hospitalizations for gout. We calculated an incidence rate of hospitalizations by dividing the number of admission episodes by person-time exposure. In tabular and graphical form, we described the incidence rate of hospitalizations over time since first gout diagnosis, using restricted cubic splines to fit a regression line.

### Treatment, urate monitoring and target attainment

For people hospitalized for gout during the study period who had a minimum of 12 months of CPRD registration after their first hospitalization, we described the number and proportion who: (i) were already prescribed ULT (allopurinol, febuxostat, benzbromarone, probenecid or sulfinpyrazone) at the time of their first hospitalization; (ii) initiated ULT within 12 months of their first hospitalization; (iii) had ≥1 SU level performed within 12 months of hospitalization; (iv) had treat-to-target urate monitoring, which we defined ≥2 SU levels within 12 months of hospitalization and/or ≥1 SU level <360 micromol/l (i.e., representing a minimum threshold for treat-to-target monitoring); and (v) had ≥1 recorded SU level <360 micromol/l or <300 micromol/l within 12 months. We described these outcomes for the hospitalized cohort as a whole, and for the subset of patients first diagnosed with gout during an admission episode. For the latter cohort, we compared attainment of these outcomes to patients first diagnosed with gout outside of an admission using two-proportions Z-tests, and described time trends in outcome attainment graphically using two-way plots.

### Factors associated with hospitalizations

We used Cox proportional hazards models with robust standard errors to describe factors associated with hospitalizations in people with incident gout. Patients were defined as at-risk from gout diagnosis until their first hospitalization, death, or date of de-registration, whichever came first. We selected covariates *a priori* on the basis of whether they were felt to be important potential confounders of hospitalizations: age at diagnosis; sex; calendar year of diagnosis; comorbidities [chronic kidney disease (CKD) stages 3–5, hypertension, diabetes mellitus, ischaemic heart disease (IHD), heart failure, previous stroke or transient ischaemic attack (TIA), obesity, current or previous history of urolithiasis]; smoking status (current/previous smoker *vs* never smoker); alcohol excess; and diuretic therapy at gout diagnosis. Further details on comorbidity definitions can be found in Supplementary Data S2, available at *Rheumatology* online. Age and sex-adjusted models and fully-adjusted models (adjusted for all covariates) were presented with hazard ratios and 95% confidence intervals. In a sensitivity analysis, adjustment was performed for baseline SU levels in patients who had these data available (baseline SU level was defined as the test closest to diagnosis, assuming this was within 6 months before/after diagnosis and not after ULT commencement). Nelson–Aalen and log-log plots were performed to ensure assumptions regarding proportional hazards were met.

Using a similar approach, we explored associations between the following factors and non-index hospitalizations: (i) ULT initiation within 12 months of diagnosis; and (ii) SU target attainment within 12 months of ULT initiation. We defined the at-risk date for hospitalizations as when ULT was initiated or SU targets were attained, respectively. For individuals who did not initiate ULT or did not attain SU targets, dummy dates were imputed using hot deck imputation to account for the greater initial risk of hospitalizations after diagnosis and ULT initiation. When exploring associations between SU target attainment and hospitalizations, we presented complete case analyses (i.e. individuals who had SU levels performed within 12 months of ULT initiation) and imputed models (i.e. all individuals who initiated ULT, with 20-cycle multiple imputation of target attainment for individuals who did not have SU levels performed within 12 months of ULT initiation). In all models, multivariable adjustment was performed for the covariates described above. As sensitivity analyses, we presented outputs from: (i) Cox proportional hazard models with inverse probability of treatment weighting of covariates; and (ii) Cox proportional hazard models including adjustment for time from diagnosis to ULT initiation.

We also explored the effect of colchicine prophylaxis when initiating ULT on hospitalizations. Individuals initiating ULT were categorized according to whether they did/did not receive ≥90 tablets of colchicine within 90 days of ULT initiation. As a sensitivity analysis, we excluded individuals who were prescribed ≥90 tablets of non-steroidal anti-inflammatory medications (NSAID: naproxen, ibuprofen, diclofenac, indomethacin, celecoxib, etoricoxib) or corticosteroids (prednisolone, prednisone, methylprednisolone, methylprednisone) within 90 days of ULT initiation.

A summary of our primary models and sensitivity analyses is included in Supplementary Table S1, available at *Rheumatology* online. All analyses were performed in Stata version 17.1.

### Study approval and ethics

The study protocol was approved by the CPRD Research Data Governance committee (approval number: 21_000680). No further ethical approval was required.

## Results

### Study population and baseline characteristics

**Figure 1.**
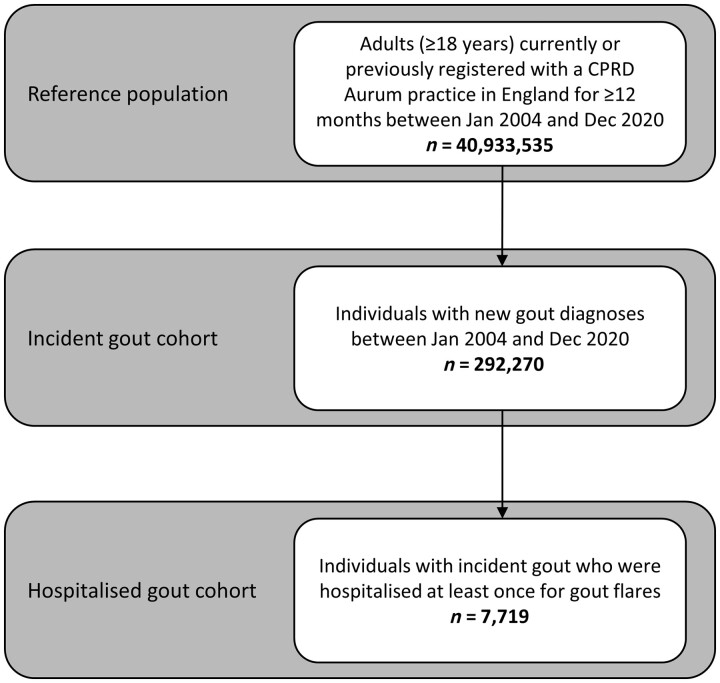
Flowchart of study populations. CPRD: Clinical Practice Research Datalink

A total of 292 270 people had new gout diagnoses in a CPRD Aurum-contributing practice in England between 1 January 2004 and 31 December 2020. From this cohort, 7719 people (2.64%) had one or more hospitalizations for gout flares during the study period, with 8920 admissions in total. A total of 6805 people (88.2%) had one admission; 914 people (11.8%) had multiple admissions. A flowchart of the study populations used in our analyses is shown in Fig. 1. The mean duration of admissions was 6 days (median: 3 days). Cumulatively, 56 857 bed-days were occupied due to gout admissions over the study period.

The baseline characteristics (at diagnosis) of people with and without hospitalizations for gout are shown in Table 1. Individuals hospitalized for gout were older, had more comorbidities, were more likely to be on diuretics, and had higher SU levels at diagnosis than those without hospitalizations.

**Table 1.** Baseline demographics and comorbidities

	All patients with gout	Patients with no admissions	Patients with one admission	Patients with multiple admissions
	*n* = 292 270	*n* = 284 551	*n* = 6805	*n* = 914
Age at diagnosis	62 (16)	61 (16)	67 (16)	66 (16)
Sex				
Male	216 630 (74.1%)	211 066 (74.2%)	4881 (71.7%)	683 (74.7%)
Number of comorbidities at diagnosis	1.6 (1.4)	1.6 (1.4)	2.3 (1.6)	2.5 (1.7)
CKD stage 3–5				
No	214 809 (73.5%)	210 617 (74.0%)	3748 (55.1%)	444 (48.6%)
Yes	77 461 (26.5%)	73 934 (26.0%)	3057 (44.9%)	470 (51.4%)
Hypertension				
No	149 422 (51.1%)	146 398 (51.4%)	2681 (39.4%)	343 (37.5%)
Yes	142 848 (48.9%)	138 153 (48.6%)	4124 (60.6%)	571 (62.5%)
Diabetes mellitus				
No	252 057 (86.2%)	246 066 (86.5%)	5309 (78.0%)	682 (74.6%)
Yes	40 213 (13.8%)	38 485 (13.5%)	1496 (22.0%)	232 (25.4%)
Ischaemic heart disease				
No	243 893 (83.4%)	238 311 (83.7%)	4957 (72.8%)	625 (68.4%)
Yes	48 377 (16.6%)	46 240 (16.3%)	1848 (27.2%)	289 (31.6%)
Heart failure				
No	271 441 (92.9%)	265 105 (93.2%)	5624 (82.6%)	712 (77.9%)
Yes	20 829 (7.1%)	19 446 (6.8%)	1181 (17.4%)	202 (22.1%)
Previous CVA				
No	273 241 (93.5%)	266 444 (93.6%)	6008 (88.3%)	789 (86.3%)
Yes	19 029 (6.5%)	18 107 (6.4%)	797 (11.7%)	125 (13.7%)
Obesity				
No	180 143 (61.6%)	175 692 (61.7%)	3947 (58.0%)	504 (55.1%)
Yes	112 127 (38.4%)	108 859 (38.3%)	2858 (42.0%)	410 (44.9%)
Urolithiasis				
No	284 996 (97.5%)	277 490 (97.5%)	6621 (97.3%)	885 (96.8%)
Yes	7274 (2.5%)	7061 (2.5%)	184 (2.7%)	29 (3.2%)
Current or ex-smoker				
No	87 442 (29.9%)	85 350 (30.0%)	1852 (27.2%)	240 (26.3%)
Yes	204 828 (70.1%)	199 201 (70.0%)	4953 (72.8%)	674 (73.7%)
Alcohol excess				
No	273 060 (93.4%)	266 041 (93.5%)	6210 (91.3%)	809 (88.5%)
Yes	19 210 (6.6%)	18 510 (6.5%)	595 (8.7%)	105 (11.5%)
On diuretic				
No	194 681 (66.6%)	190 924 (67.1%)	3346 (49.2%)	411 (45.0%)
Yes	97 589 (33.4%)	93 627 (32.9%)	3459 (50.8%)	503 (55.0%)
Serum urate at diagnosis, micromol/L	471 (100)	470 (99)	523 (117)	558 (126)

Baseline demographics and comorbidities in people with newly-diagnosed gout, separated into those who had hospitalizations for gout flares (single *vs* multiple) during the study period and those who had no hospitalizations. Data are presented as mean (s.d.) for continuous measures, and *n* (%) for categorical measures.

Baseline serum urate levels were available for 184 185 patients.

CKD: chronic kidney disease; CVA: cerebrovascular accident.

Of 8920 admissions, 713 (7.99%) occurred in patients after prior attainment of a SU level <360 micromol/l, while 325 admissions (3.64%) occurred after prior attainment of a SU level <300 micromol/l.

### Incidence rate of hospitalizations

Of 8920 admissions, 3316 (37.2%) were index diagnosis events (i.e. first recorded diagnosis of gout made during a hospitalization for flare), while 5604 occurred ≥7 days after first diagnosis.

**Figure 2.**
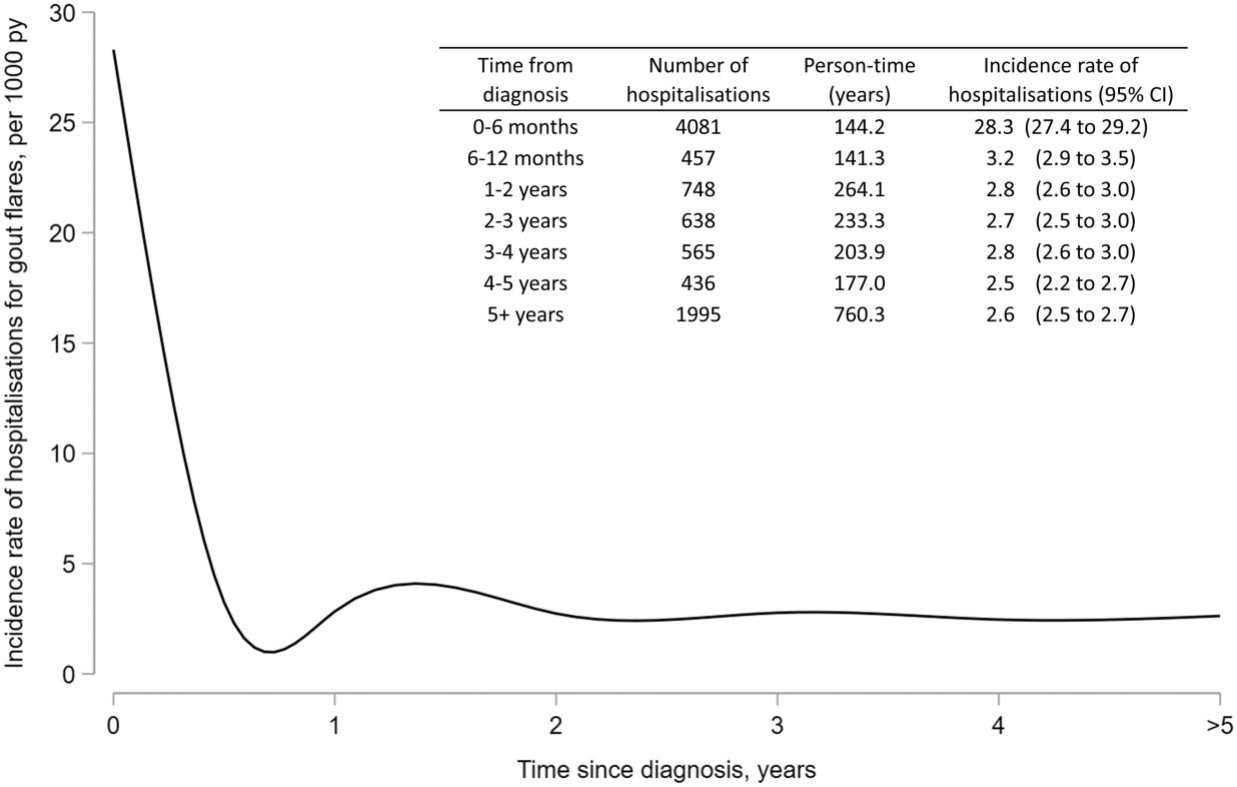
Incidence rate of hospitalizations for flares in people with gout, in relation to time since diagnosis. Restricted cubic splines were used to fit a regression line. PY: person-years

The incidence rate of hospitalizations for flares in people with gout was 4.64 per 1000 person-years (95% CI 4.54, 4.73). The incidence rate of hospitalizations was greater within 6 months of diagnosis (28.3 admissions per 1000 person-years; 95% CI 27.4, 29.2) than beyond 6 months (2.71 admissions per 1000 person-years; 95% CI 2.64, 2.80), as shown in Fig. 2.

### ULT initiation and urate target attainment

Of 7719 patients hospitalized for gout, 7040 (91.2%) had ≥12 months of available follow-up after their first hospitalization, to facilitate analyses of post-discharge ULT initiation and SU target attainment. A total of 1734/7040 people (24.6%) were already prescribed ULT at the time of their first hospitalization; 2560 (36.4%) commenced ULT within 12 months of hospitalization; and 2746 (39.0%) remained off ULT 12 months after their first hospitalization.

In total, 3360/7040 people (47.7%) had ≥1 SU level performed within 12 months of their first hospitalization; 1956 (27.8%) had treat-to-target urate monitoring. Of 3360 hospitalized patients who had ≥1 SU level performed, 1184 (35.2%) attained a SU <360 micromol/l within 12 months, while 581 (17.3%) attained a SU <300 micromol/l.

**Figure 3.**
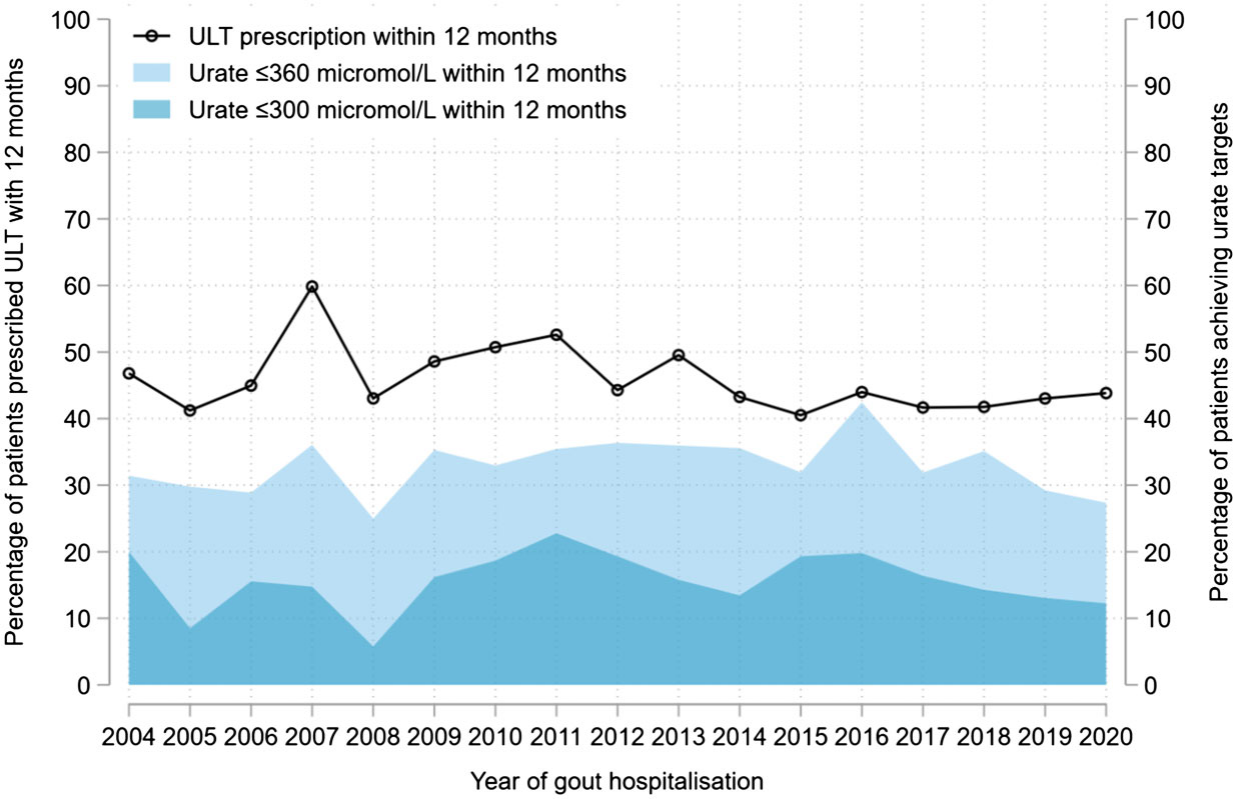
Trends in ULT initiation and urate target attainment following new gout diagnoses made during hospitalizations. Time trends in the proportion of patients newly diagnosed with gout during hospitalizations for flares (n** **=** **3316) who: (i) were initiated on urate-lowering therapy (ULT) within 12 months of hospitalization (black line); or (ii) had a SU performed (*n*** **=** **1529) and attained a level ≤360 µmol/L (light blue) or ≤300 µmol/L (dark blue) within 12 months of hospitalization

Of the subset of patients first diagnosed with gout during hospitalizations for flares (*n* = 3316), 1504 (45.4%) were prescribed ULT within 12 months of diagnosis. In comparison, people diagnosed with gout outside of a hospitalization (*n* = 288 954) were less likely to initiate ULT within 12 months of diagnosis (27.9%; *P* < 0.001). People first diagnosed with gout during a hospitalization were also more likely to receive treat-to-target urate monitoring (25.7% *vs* 22.0%, respectively; *P* < 0.001) and to attain SU levels <300 micromol/l (15.8% *vs* 13.2%; *P* < 0.001) and <360 micromol/l (33.4% *vs* 27.4%; *P* < 0.001) within 12 months of diagnosis than those diagnosed outside of an admission. Time trends in post-discharge ULT initiation and SU target attainment are shown in Fig. 3.

### Baseline factors associated with hospitalizations

In Cox proportional hazard models with multivariable adjustment, the following factors at diagnosis associated with hospitalizations for flares in people with gout: older age, male sex, diuretic use, comorbidities (CKD, heart failure, alcohol excess, IHD, diabetes mellitus, previous CVA and obesity), and later calendar year of diagnosis (Table 2). Following adjustment for SU level at diagnosis in the subset of patients who had levels performed (*n* = 184 185), these variables remained significant predictors of hospitalizations, albeit with a reduced effect size for several comorbidities (Supplementary Table S2, available at *Rheumatology* online).

**Table 2.** Factors associated with hospitalizations for flares in people with gout

Variables	Hazard ratio (age/sex-adjusted)	95% CI	*P*-value	Hazard ratio (fully adjusted)	95% CI	*P*-value
Age at diagnosis (per 10-year increase)	1.32	(1.29, 1.34)	<0.001	1.10	(1.08, 1.13)	<0.001
Female sex	0.90	(0.85, 0.94)	<0.001	0.82	(0.78, 0.86)	<0.001
Later calendar year of diagnosis	1.02	(1.02, 1.03)	<0.001	1.03	(1.02, 1.03)	<0.001
CKD stages 3–5	2.03	(1.91, 2.14)	<0.001	1.68	(1.59, 1.79)	<0.001
Hypertension	1.24	(1.18, 1.31)	<0.001	0.96	(0.91, 1.02)	0.19
Diabetes mellitus	1.67	(1.58, 1.76)	<0.001	1.32	(1.25, 1.39)	<0.001
Ischaemic heart disease	1.58	(1.50, 1.67)	<0.001	1.15	(1.09, 1.22)	<0.001
Heart failure	2.66	(2.50, 2.83)	<0.001	1.89	(1.77, 2.02)	<0.001
Previous CVA	1.62	(1.51, 1.73)	<0.001	1.37	(1.28, 1.47)	<0.001
Urolithiasis	1.05	(0.92, 1.21)	0.46	0.98	(0.85, 1.13)	0.77
Obesity	1.27	(1.21, 1.33)	<0.001	1.11	(1.06, 1.17)	<0.001
Current/ex-smoker	1.08	(1.03, 1.14)	<0.001	0.97	(0.92, 1.02)	0.19
Alcohol excess	1.74	(1.60, 1.89)	<0.001	1.72	(1.59, 1.87)	<0.001
Diuretic therapy	1.71	(1.63, 1.80)	<0.001	1.33	(1.25, 1.42)	<0.001

Age and sex-adjusted Cox proportional hazard model outputs are shown, in addition to multivariable Cox proportional hazard model outputs (with adjustment for all covariates, including calendar year of diagnosis). Robust standard errors were estimated to account for clustering of patients within practice/region.

CKD: chronic kidney disease; CVA: cerebrovascular accident.

### Associations between ULT initiation and hospitalizations

**Figure 4.**
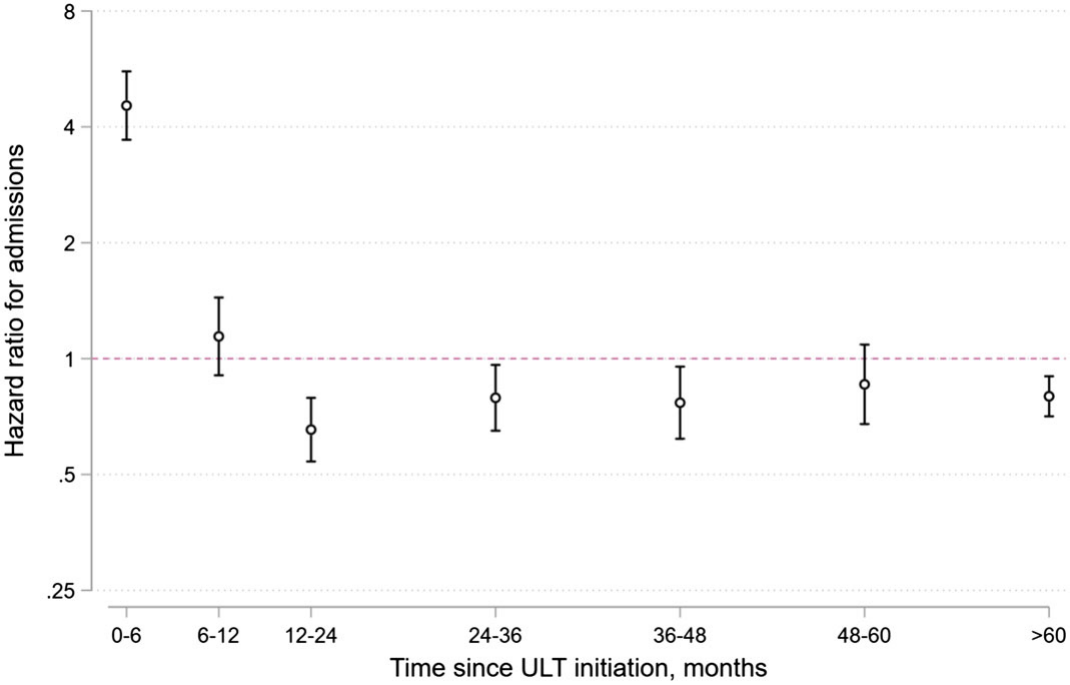
Risk of hospitalization for flares in people with gout who initiated urate-lowering therapy (ULT) within 12 months of diagnosis, compared with people who did not initiate ULT. Outputs from Cox proportional hazards models are shown, highlighting the change in hazard ratio for hospitalizations in relation to time elapsed following initiation of ULT. Adjustment was performed for the following covariates: age, sex, calendar year of gout diagnosis, diuretic use and comorbidities at diagnosis (hypertension, CKD, IHD, heart failure, diabetes mellitus, prior CVA, obesity, smoking status, alcohol excess, history of urolithiasis). A logarithmic y-axis was used, to reflect the exponential distribution of hazard functions

In Cox proportional hazard models with multivariable adjustment, an increased risk of hospitalizations for flares was observed within the first 6 months of initiating ULT, compared with people with gout who did not initiate ULT: adjusted hazard ratio (aHR) 4.54; 95% CI 3.70, 5.58; *P* < 0.001. Between 6 and 12 months after ULT initiation, there was no significant association with hospitalizations (aHR 1.14; 95% CI 0.91, 1.44; *P* = 0.26). Beyond 12 months after ULT initiation, there was a reduced risk of hospitalizations associated with ULT initiation (aHR 0.77; 95% CI 0.71, 0.83; *P* < 0.001), when compared with patients who did not initiate ULT (Fig. 4).

Following adjustment for SU level at diagnosis in the subset of patients who had these levels performed (*n* = 184 185), the association between ULT and increased hospitalizations within 6 months of initiation remained but reduced in effect size (aHR 3.15; 95% CI 2.41, 4.12; *P* < 0.001), while the association between ULT and fewer hospitalizations beyond 12 months increased in effect size (aHR 0.63; 95% CI 0.57, 0.70; *P* < 0.001) (Supplementary Fig. S1, available at *Rheumatology* online). In sensitivity analyses comparing our primary Cox model to a propensity model with inverse probability of treatment weighting, the results were very similar (Supplementary Fig. S2, available at *Rheumatology* online), as were Cox models that included adjustment for time from diagnosis to ULT initiation (Supplementary Figs S3 and S4, available at *Rheumatology* online).

We explored whether prescription of colchicine prophylaxis during ULT initiation impacted upon hospitalization risk. Of 81 994 people initiating ULT, 8026 (9.8%) received ≥90 tablets of colchicine in the 3 months after ULT initiation. In age and sex-adjusted Cox models, there was an associated increased risk of hospitalizations within 6 months after ULT initiation in people prescribed *vs* not prescribed colchicine prophylaxis (HR 1.41; 95% CI 1.02, 1.94; *P* = 0.038). In fully-adjusted Cox models, however, there were no statistically significant differences between these groups (aHR 1.31; 95% CI 0.95, 1.82; *P* = 0.10). In sensitivity analyses excluding individuals prescribed NSAID prophylaxis or corticosteroid prophylaxis (*n* = 9559), colchicine prophylaxis did not associate with significant differences in hospitalizations within 6 months of ULT initiation (aHR 1.32; 95% CI 0.93, 1.87; *P* = 0.12).

### Associations between urate target attainment and hospitalizations

Finally, we investigated associations between SU target attainment in people initiating ULT (*n* = 81 994) and hospitalizations. Using Cox proportional hazards with multiple imputation for people with no SU levels performed within 12 months of ULT initiation (*n* = 36 704), attainment of a SU <360 micromol/l associated with a reduced risk of hospitalizations after target attainment (aHR 0.57; 95% CI 0.49, 0.67; *P* < 0.001) when compared with people initiating ULT but not attaining target. For those attaining a SU <300 micromol/l, the adjusted hazard ratio for hospitalizations was 0.69 (95% CI 0.57, 0.84; *P* < 0.001). In complete case analyses—restricted to people initiating ULT who had ≥1 SU level performed within 12 months of initiation (*n* = 45 290)—the hazard ratios for hospitalizations were 0.39 (95% CI 0.32, 0.47; *P* < 0.001) for attaining <360 micromol/l and 0.47 (95% CI 0.37, 0.59; *P* < 0.001) for attaining <300 micromol/l. Similar findings were observed in propensity models with inverse probability of treatment weighting: <360 micromol/l (HR 0.39; 95% CI 0.32, 0.47; *P* < 0.001) and <300 micromol/l (HR 0.48; 95% CI 0.37, 0.61; *P* < 0.001).

## Discussion

In this study, we described the incidence of hospitalizations for gout and the impact of treat-to-target ULT in over 290 000 people with gout. We observed an increased risk of hospitalizations in the first 6 months after ULT initiation, and a reduced risk of hospitalizations beyond 12 months. In people initiating ULT, attainment of target SU levels associated with a 30–60% lower risk of hospitalizations for flares. Despite this, only a third of patients achieved a SU target within a year of hospitalization.

Previous studies from the US, UK and Europe have used aggregated health data to demonstrate large increases in hospitalizations for gout over the last 20–30 years [1, 5–7]. Our study is the first to use individual-level, linked primary and secondary care data to describe the incidence and pattern of hospitalizations in a nationwide cohort of incident gout patients. For every 1000 people with gout, there were 4.6 hospitalizations with primary diagnoses of gout per year between 2004 and 2020. There was a 10-fold increased incidence of hospitalizations during the first 6 months of diagnosis. Older patients, those with comorbidities, diuretic users and people with higher SU levels at diagnosis were most at risk of being hospitalized.

Previously, two small retrospective analyses (≤250 patients each) reported associations between ULT and reduced risks of hospitalizations or ED attendances for gout [13, 14]. The time-varying relationship between ULT and hospitalizations, and the impact of achieving SU targets, were not known. Our finding that SU target attainment after ULT initiation associates with fewer hospitalizations demonstrates the importance of treat-to-target ULT in the long-term prevention of admissions. Hospitalizations with primary diagnoses of gout cost the English NHS more than £10 million per year [15]. Additional costs are attributable to ED attendances, hospitalizations with secondary diagnoses of gout (e.g. in the context of heart failure), repeated primary care attendances, and work disability due to flares [16, 17]. In our study, 63% of admissions occurred in people already diagnosed with gout; however, only 25% of admitted patients were receiving ULT; 40% remained on no ULT at 12 months after their first hospitalization; and only a third of patients achieved a SU <360 micromol/l within 12 months. Despite the publication of British, European and American guidelines that encourage treat-to-target ULT [18–20], we observed no improvements in ULT initiation or urate target attainment between January 2004 and December 2020. Together, these findings emphasize the need for implementation strategies that promote the uptake of treat-to-target ULT, particularly for patients most at risk of hospitalization.

Our finding that ULT associates with an increased risk of flares requiring hospitalization in the first 6 months after initiation is in keeping with the results of studies in community settings. In a UK primary care-based RCT of people with gout (*n* = 517), treat-to-target ULT increased the frequency of gout flares within the first year when compared with usual care, but reduced flares at 2 years [9]. In the NOR-Gout study of treat-to-target ULT, flares were more frequent during the first year after initiation (particularly at 3–6 months after initiation), but reduced greatly in the second year [21].

Changes in SU levels when initiating ULT may precipitate flares through dissolution and remodelling of intra-articular urate crystal deposits [22]. Guidelines recommend considering prescription of prophylaxis against flares when initiating and titrating ULT, with low-dose colchicine (500 micrograms once or twice daily for ≥3 months) recommended as first-line prophylaxis [18, 19]. In our cohort, only 10% of people initiating ULT were prescribed the equivalent of colchicine 500 micrograms once daily for ≥3 months. In age and sex-adjusted models, we observed an association between increased hospitalizations and the prescription of colchicine prophylaxis; however, this association was not statistically significant following multivariable adjustment. Our finding contrasts RCTs that have reported fewer flares when initiating ULT with colchicine prophylaxis [23]. The differences may represent confounding by indication in our cohort; for example, prescription of colchicine to people with more severe gout at greater risk of hospitalization. We explored the use of propensity models to account for differences in colchicine-receiving *vs* non-receiving groups; however, differences between these groups precluded this. Other potential contributing factors could include repeated acute prescriptions for colchicine for flares being misclassified as prophylaxis, and low adherence to prophylaxis during ULT titration.

Our study had several strengths. We used validated, population-level data sources containing pseudonymised data on 41 million people, covering a period of 17 years [24–26]. Linked secondary care data on all admissions to NHS hospitals in England were available for 98% of the study cohort, facilitating accurate estimates of hospitalizations. We used several statistical approaches to explore identified associations, including propensity models, and accounted for multiple possible confounders.

Our study also had limitations. There is a potential for diagnostic misclassification inherent to studies using coded healthcare data. We defined hospitalizations for gout flares as admissions with primary diagnoses of gout using the ICD-10 coding system. Although most primary admissions for gout will have been due to flares, other factors may have contributed to these admissions; for example, associations between gout flares and cardiovascular events were recently reported [27]. We were unable to infer the directionality of reported associations. Reverse causality may have contributed to the increased risk of hospitalizations observed within 6 months of ULT initiation; supported by our finding that people first diagnosed with gout during an admission were 65% more likely to be prescribed ULT than those diagnosed outside of an admission. Additionally, our analyses do not take into account the impact of medication adherence or persistence on outcomes.

We did not include ED attendances or secondary admission diagnoses of gout, due to the less granular/reliable coding of these episodes [12]. This will have substantially underestimated the true burden of gout, noting that 76% of unplanned hospital attendances for gout in a recent UK-based study were ED attendances that did not require admission [28]. Furthermore, as our analyses were performed in a cohort of incidence gout patients in England, the findings are not necessarily generalizable to other healthcare services or to people with longstanding gout.

In conclusion, the prescription of ULT in people with gout associates with an increased risk of hospitalizations for flares within the first 6 months of initiation, but reduces hospitalizations from 12 months onwards particularly when SU targets are achieved. Despite this, only a third of patients achieved SU targets within a year of discharge from hospital, and 40% remained on no ULT. If avoidable admissions are to be prevented in the long term, treat-to-target ULT must be implemented.

## Supplementary Material

keac638_Supplementary_Data

## Data Availability

The anonymised, coded data used in these analyses were provided by CPRD following approval by their Research Data Governance committee. The data are available on request from CPRD. Additional code lists used in these analyses are available on request from the corresponding author.
