# Supplementary material for: Treat-to-target urate-lowering therapy and hospitalizations for gout: results from a nationwide cohort study in England
Source: Rheumatology (Oxford). 2022 Nov 10;62(7):2426–34. doi: 10.1093/rheumatology/keac638 (PMC10321109; doi:10.1093/rheumatology/keac638)
Supplement: keac638_Supplementary_Data [file keac638_supplementary_data.pdf]

Supplementary Table S1. Summary of prediction models used in the study.

Study population	Exposure group	Comparison group	Primary model	At-risk date	Covariates	Sensitivity analyses
People with incident gout	People with gout who were hospitalised for gout flares	People with gout who were not hospitalised for gout flares	Cox proportional hazards	From gout diagnosis	Age, sex, calendar year of diagnosis, CKD, hypertension, diabetes mellitus, IHD, heart failure, previous CVA, obesity, urolithiasis; smoking status, alcohol excess, diuretic therapy	1. Adjustment for serum urate level at diagnosis
People with incident gout	ULT initiated within 12 months of diagnosis	No ULT initiated within 12 months of diagnosis	Cox proportional hazards	From ULT initiation (dummy date imputed for ULT non-initiators)		1. Adjustment for serum urate level at diagnosis<br>2. Propensity model with IPTW<br>3. Adjustment for time from diagnosis to ULT initiation
People with gout who initiated ULT	Colchicine prophylaxis prescribed for ≥3 months	Colchicine prophylaxis not prescribed for ≥3 months	Cox proportional hazards	From ULT initiation		1. Exclusion of individuals prescribed NSAID or corticosteroid prophylaxis
People with gout who initiated ULT	Urate <360 micromol/L achieved within 12 months of ULT initiation	Urate <360 micromol/L not achieved within 12 months of ULT initiation	Cox proportional hazards with multiple imputation of target attainment in those without urate levels	From target attainment (dummy date imputed for target non-attainers)		1. Complete case analysis (unimputed)<br>2. Propensity model with IPTW
People with gout who initiated ULT	Urate <300 micromol/L achieved within 12 months of ULT initiation	Urate <300 micromol/L not achieved within 12 months of ULT initiation	Cox proportional hazards with multiple imputation of target attainment in those without urate levels	From target attainment (dummy date imputed for target non-attainers)		1. Complete case analysis (unimputed)<br>2. Propensity model with IPTW

ULT: urate-lowering therapy; CKD: chronic kidney disease; IHD: ischaemic heart disease; CVA: cerebrovascular accident; IPTW: inverse probability of treatment weighting; NSAID: non-steroidal anti-inflammatory drug.

Supplementary Table S2. Factors associated with hospitalisations for flares in people with gout who had baseline serum urate data available (n=184,185).

	Without adjustment for baseline urate			With adjustment for baseline urate		
**Variables**	**Hazard ratio**	**95% CI**	**p-value**	**Hazard ratio**	**95% CI**	**p-value**
Age at diagnosis (per 10-year increase)	1.11	(1.07 - 1.15)	<0.001	1.15	(1.11 - 1.19)	<0.001
Female sex	0.76	(0.71 - 0.82)	<0.001	0.83	(0.77 - 0.89)	<0.001
Year of gout diagnosis	1.03	(1.02 - 1.04)	<0.001	1.03	(1.02 - 1.04)	<0.001
CKD stages 3-5	1.67	(1.53 - 1.81)	<0.001	1.43	(1.31 - 1.56)	<0.001
Hypertension	0.99	(0.91 - 1.08)	0.86	1.00	(0.92 - 1.08)	0.97
Diabetes mellitus	1.22	(1.13 - 1.33)	<0.001	1.23	(1.14 - 1.34)	<0.001
Ischaemic heart disease	1.15	(1.06 - 1.25)	<0.001	1.15	(1.06 - 1.25)	<0.001
Heart failure	2.05	(1.87 - 2.26)	<0.001	1.89	(1.71 - 2.08)	<0.001
Previous CVA	1.31	(1.18 - 1.45)	<0.001	1.30	(1.17 - 1.44)	<0.001
Urolithiasis	1.00	(0.83 - 1.22)	0.97	1.00	(0.83 - 1.22)	0.97
Obesity	1.13	(1.05 - 1.21)	<0.001	1.08	(1.01 - 1.16)	0.03
Current/ex-smoker	0.95	(0.88 - 1.02)	0.14	0.94	(0.88 - 1.01)	0.11
Alcohol excess	1.74	(1.54 - 1.96)	<0.001	1.68	(1.49 - 1.90)	<0.001
Diuretic therapy	1.30	(1.20 - 1.42)	<0.001	1.12	(1.03 - 1.22)	0.01
Baseline serum urate level (>480 micromol/L)	-	-	-	1.90	(1.77 - 2.05)	<0.001

Multivariable Cox proportional hazard model outputs are shown, with (right-hand side) and without (left-hand side) adjustment for serum urate level at diagnosis. Serum urate was included as a binary variable (above/below 480 micromol/L) corresponding to the threshold specified in the EULAR gout management guideline. Outputs were adjusted for all other covariates shown, including calendar year of diagnosis. Robust standard errors were estimated to account for clustering of patients within practice/region. CKD: chronic kidney disease; CVA: cerebrovascular accident.

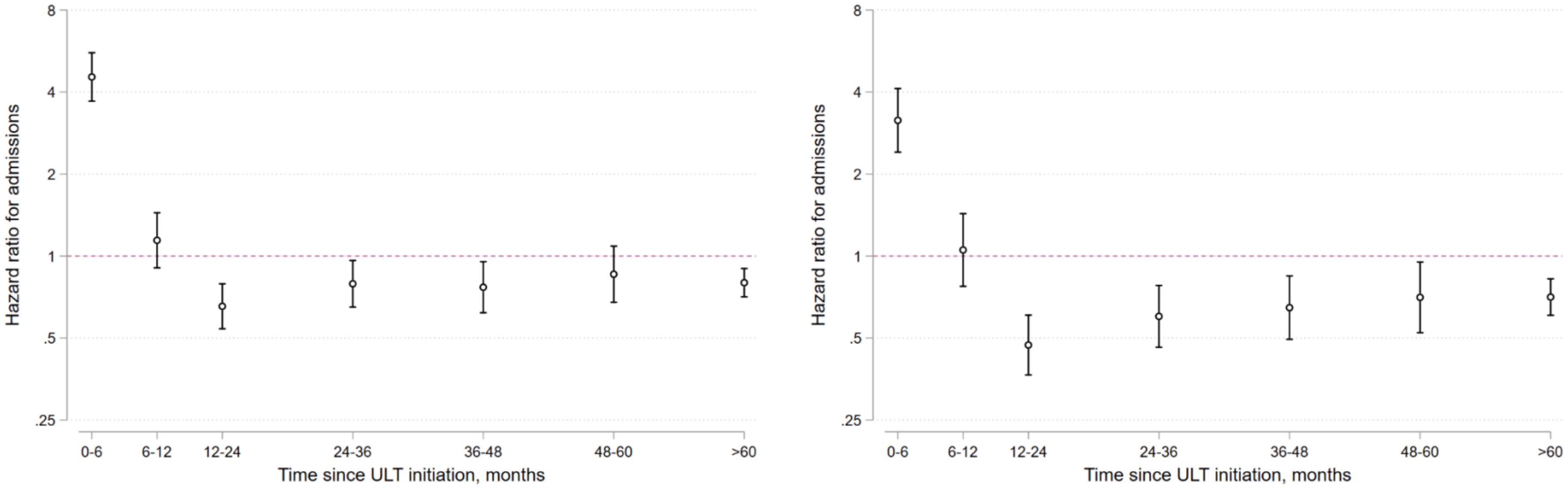

Supplementary Figure S1. Hazard ratio of hospitalisations for flares in people with gout who initiated urate-lowering therapy (ULT) within 12 months of diagnosis, relative to those who did not initiate ULT. Outputs are shown from Cox proportional hazard models: i) without adjustment for serum urate levels at diagnosis (left panel; primary model); and ii) with adjustment for serum urate levels at diagnosis (right panel; sensitivity analysis). In both models, the following covariates were adjusted for: age, sex, calendar year of gout diagnosis, diuretic use and comorbidities at diagnosis (hypertension, CKD, IHD, heart failure, diabetes mellitus, prior CVA, obesity, smoking status, alcohol excess, history of urolithiasis). A logarithmic y-axis was used, to reflect the exponential distribution of hazard functions.

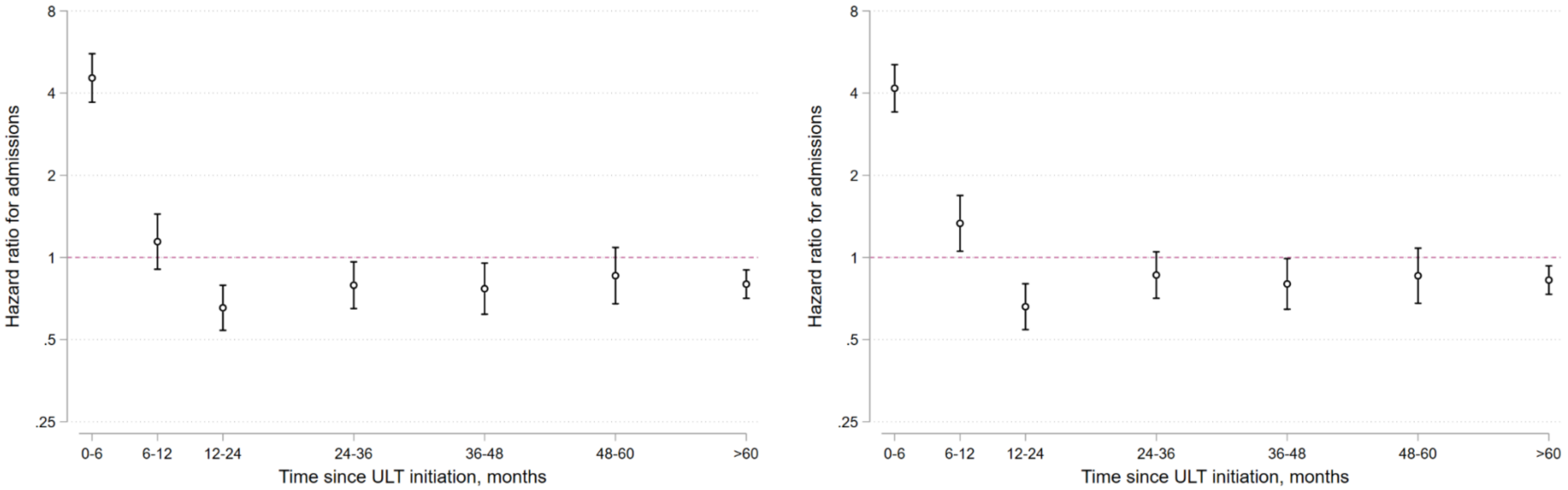

Supplementary Figure S2. Hazard ratio of hospitalisations for flares in people with gout who initiated urate-lowering therapy (ULT) within 12 months of diagnosis, relative to those who did not initiate ULT. Outputs from our primary Cox proportional hazard model (left panel) are compared to a propensity model with inverse probability treatment weighting (right panel). In both models, adjustment/weighting was performed for the following covariates: age, sex, calendar year of gout diagnosis, diuretic use and comorbidities at diagnosis (hypertension, CKD, IHD, heart failure, diabetes mellitus, prior CVA, obesity, smoking status, alcohol excess, history of urolithiasis). A logarithmic y-axis was used, to reflect the exponential distribution of hazard functions.

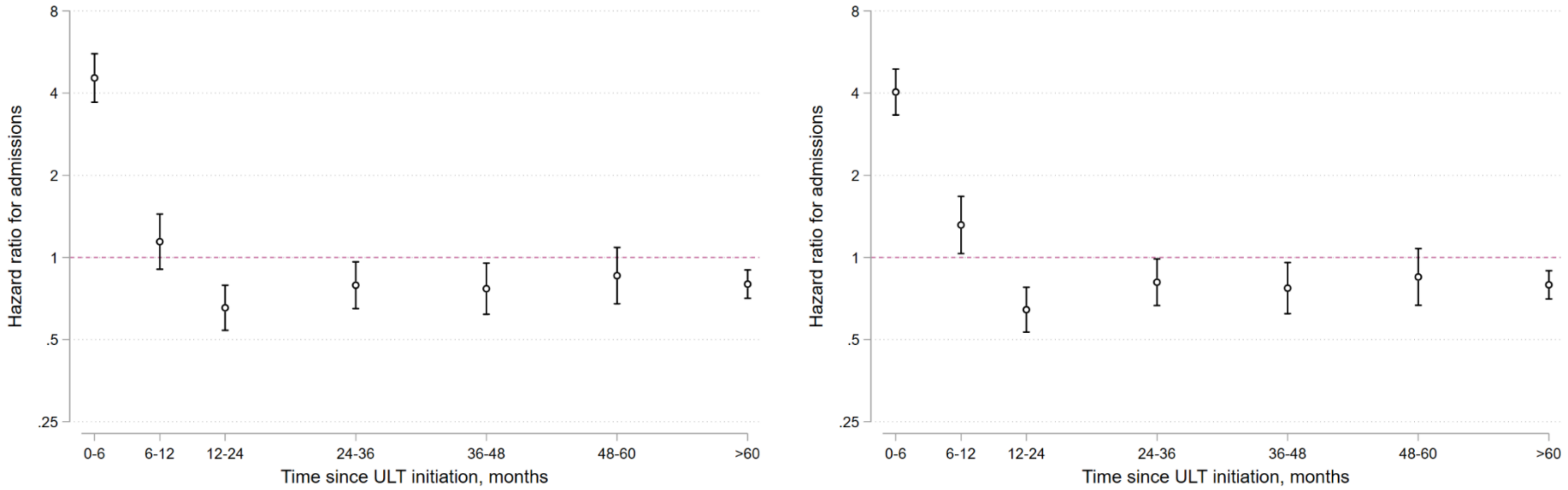

Supplementary Figure S3. Hazard ratio of hospitalisations for flares in people with gout who initiated urate-lowering therapy (ULT) within 12 months of diagnosis, relative to those who did not initiate ULT. Outputs from our primary Cox proportional hazard model (left panel) are compared to a sensitivity analysis that included adjustment for time from gout diagnosis to ULT initiation (right panel). A histogram of time from gout diagnosis to first prescription of ULT is shown in Supplementary Figure S4. In both models, adjustment/weighting was performed for the following covariates: age, sex, calendar year of gout diagnosis, diuretic use and comorbidities at diagnosis (hypertension, CKD, IHD, heart failure, diabetes mellitus, prior CVA, obesity, smoking status, alcohol excess, history of urolithiasis). A logarithmic y-axis was used, to reflect the exponential distribution of hazard functions.

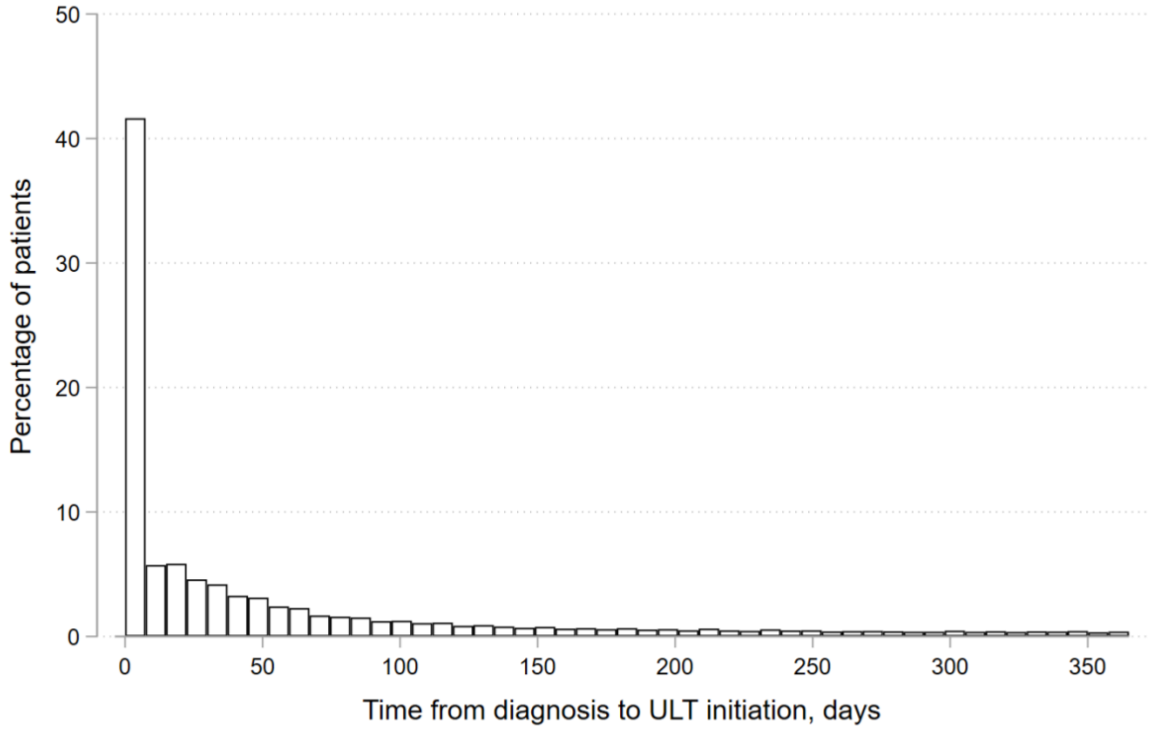

Supplementary Figure S4. Histogram of time from gout diagnosis to first prescription of ULT in patients who initiated ULT within 12 months of diagnosis. ULT: urate-lowering therapy.

Supplementary Data S1. Gout diagnosis SNOMED codes

924311000000106	Acute exacerbation of gout
24595009	Acute gouty arthritis
24595009	Acute gouty arthropathy
48440001	Arthritis due to gout
16068491000119107	Arthritis of bilateral knees due to gout
67148009	Arthritis of great toe due to gout
1074021000119103	Arthritis of left foot due to gout
1074031000119100	Arthritis of left great toe due to gout
1074001000119107	Arthritis of right foot due to gout
1074011000119105	Arthritis of right great toe due to gout
428839004	Arthritis of toe due to gout
48440001	Articular gout
16040031000119108	Bilateral chronic tophaceous gout of elbows
16040071000119106	Bilateral chronic tophaceous gout of hands
16015911000119102	Bilateral gouty arthritis of ankles
16015951000119101	Bilateral gouty arthritis of feet
16010751000119107	Bilateral gouty arthritis of great toes
16068491000119107	Bilateral gouty arthritis of knees
16005991000119109	Bilateral gouty tophus of elbows
16076011000119100	Bilateral gouty tophus of feet
16006631000119109	Bilateral gouty tophus of hands
68451005	Chronic arthritis due to gout
306141000119109	Chronic gout of ankle and/or foot without tophus caused by drug
306141000119109	Chronic gout of ankle and/or foot without tophus due to drug
303891000119104	Chronic gout of ankle without tophus due to renal impairment
306161000119108	Chronic gout of elbow without tophus caused by drug
306161000119108	Chronic gout of elbow without tophus due to drug
303911000119102	Chronic gout of elbow without tophus due to renal impairment
306181000119104	Chronic gout of hand without tophus caused by drug
306181000119104	Chronic gout of hand without tophus due to drug

303931000119107 Chronic gout of hand without tophus due to renal impairment

306201000119103 Chronic gout of hip without tophus caused by drug

306201000119103 Chronic gout of hip without tophus due to drug

303951000119101 Chronic gout of hip without tophus due to renal impairment

306221000119107 Chronic gout of knee without tophus caused by drug

306221000119107 Chronic gout of knee without tophus due to drug

303971000119105 Chronic gout of knee without tophus due to renal impairment

306381000119102 Chronic gout of multiple sites without tophus caused by drug

306381000119102 Chronic gout of multiple sites without tophus due to drug

304131000119106 Chronic gout of multiple sites without tophus due to renal impairment

306541000119104 Chronic gout of shoulder without tophus caused by drug

306541000119104 Chronic gout of shoulder without tophus due to drug

304291000119102 Chronic gout of shoulder without tophus due to renal impairment

306561000119100 Chronic gout of vertebra without tophus caused by drug

306561000119100 Chronic gout of vertebra without tophus due to drug

304311000119103 Chronic gout of vertebra without tophus due to renal impairment

306601000119100 Chronic gout of wrist without tophus caused by drug

306601000119100 Chronic gout of wrist without tophus due to drug

304351000119102 Chronic gout of wrist without tophus due to renal impairment

710733002 Chronic gout without tophus

306581000119109 Chronic gout without tophus caused by drug

306581000119109 Chronic gout without tophus due to drug

304331000119108 Chronic gout without tophus due to renal impairment

68451005 Chronic gouty arthritis

190829000 Chronic gouty nephropathy

721285007 Chronic primary gouty arthritis

73877009 Chronic tophaceous gout

306571000119106 Chronic tophaceous gout caused by drug

306571000119106 Chronic tophaceous gout due to drug

304321000119105 Chronic tophaceous gout due to renal impairment

306131000119100 Chronic tophaceous gout of ankle and/or foot caused by drug

306131000119100	Chronic tophaceous gout of ankle and/or foot due to drug
303881000119102	Chronic tophaceous gout of ankle due to renal impairment
16040031000119108	Chronic tophaceous gout of bilateral elbows
16040071000119106	Chronic tophaceous gout of bilateral hands
16040031000119108	Chronic tophaceous gout of both elbows
16040071000119106	Chronic tophaceous gout of both hands
306151000119106	Chronic tophaceous gout of elbow caused by drug
306151000119106	Chronic tophaceous gout of elbow due to drug
303901000119100	Chronic tophaceous gout of elbow due to renal impairment
306171000119102	Chronic tophaceous gout of hand caused by drug
306171000119102	Chronic tophaceous gout of hand due to drug
303921000119109	Chronic tophaceous gout of hand due to renal impairment
306191000119101	Chronic tophaceous gout of hip caused by drug
306191000119101	Chronic tophaceous gout of hip due to drug
303941000119103	Chronic tophaceous gout of hip due to renal impairment
306211000119100	Chronic tophaceous gout of knee caused by drug
306211000119100	Chronic tophaceous gout of knee due to drug
303961000119104	Chronic tophaceous gout of knee due to renal impairment
306371000119100	Chronic tophaceous gout of multiple sites caused by drug
306371000119100	Chronic tophaceous gout of multiple sites due to drug
304121000119108	Chronic tophaceous gout of multiple sites due to renal impairment
306531000119108	Chronic tophaceous gout of shoulder caused by drug
306531000119108	Chronic tophaceous gout of shoulder due to drug
304281000119100	Chronic tophaceous gout of shoulder due to renal impairment
306551000119102	Chronic tophaceous gout of vertebra caused by drug
306551000119102	Chronic tophaceous gout of vertebra due to drug
304301000119101	Chronic tophaceous gout of vertebra due to renal impairment
306591000119107	Chronic tophaceous gout of wrist caused by drug
306591000119107	Chronic tophaceous gout of wrist due to drug
304341000119104	Chronic tophaceous gout of wrist due to renal impairment
170735000	Date gout treatment started

306141000119109	Drug induced chronic gout of ankle and/or foot without tophus
306161000119108	Drug induced chronic gout of elbow without tophus
306181000119104	Drug induced chronic gout of hand without tophus
306201000119103	Drug induced chronic gout of hip without tophus
306221000119107	Drug induced chronic gout of knee without tophus
306381000119102	Drug induced chronic gout of multiple sites without tophus
306541000119104	Drug induced chronic gout of shoulder without tophus
306561000119100	Drug induced chronic gout of vertebra without tophus
306601000119100	Drug induced chronic gout of wrist without tophus
306581000119109	Drug induced chronic gout without tophus
306571000119106	Drug induced chronic tophaceous gout
306131000119100	Drug induced chronic tophaceous gout of ankle and/or foot
306151000119106	Drug induced chronic tophaceous gout of elbow
306171000119102	Drug induced chronic tophaceous gout of hand
306191000119101	Drug induced chronic tophaceous gout of hip
306211000119100	Drug induced chronic tophaceous gout of knee
306371000119100	Drug induced chronic tophaceous gout of multiple sites
306531000119108	Drug induced chronic tophaceous gout of shoulder
306551000119102	Drug induced chronic tophaceous gout of vertebra
306591000119107	Drug induced chronic tophaceous gout of wrist
306611000119102	Drug induced gout of ankle and/or foot
306621000119109	Drug induced gout of elbow
306631000119107	Drug induced gout of hand
306641000119103	Drug induced gout of hip
306651000119101	Drug induced gout of knee
306731000119104	Drug induced gout of multiple sites
306811000119101	Drug induced gout of shoulder
306821000119108	Drug induced gout of vertebra
306831000119106	Drug induced gout of wrist
239845005	Drug-induced gout
330060000	Drugs for the treatment of gout

46785007	Familial gout with renal failure
46785007	Familial juvenile gout
90560007	Gout
170737008	Gout associated problem
170737008	Gout associated problem
170737008	Gout associated problems
239845005	Gout caused by drug
239846006	Gout caused by lead
239844009	Gout due to impairment of renal function
239846006	Gout due to lead poisoning
90560007	Gout NOS
646651000000108	Gout NOS
306611000119102	Gout of ankle and/or foot caused by drug
306611000119102	Gout of ankle and/or foot due to drug
306621000119109	Gout of elbow caused by drug
306621000119109	Gout of elbow due to drug
308791000119101	Gout of elbow due to renal impairment
306631000119107	Gout of hand caused by drug
306631000119107	Gout of hand due to drug
308801000119100	Gout of hand due to renal impairment
306641000119103	Gout of hip caused by drug
306641000119103	Gout of hip due to drug
308811000119102	Gout of hip due to renal impairment
306651000119101	Gout of knee caused by drug
306651000119101	Gout of knee due to drug
308821000119109	Gout of knee due to renal impairment
306731000119104	Gout of multiple sites caused by drug
306731000119104	Gout of multiple sites due to drug
308901000119105	Gout of multiple sites due to renal impairment
306811000119101	Gout of shoulder caused by drug
306811000119101	Gout of shoulder due to drug

298941000119101	Gout of shoulder due to renal impairment
306821000119108	Gout of vertebra caused by drug
306821000119108	Gout of vertebra due to drug
298951000119104	Gout of vertebra due to renal impairment
306831000119106	Gout of wrist caused by drug
306831000119106	Gout of wrist due to drug
298961000119102	Gout of wrist due to renal impairment
239845005	Gout secondary to drug
239847002	Gout secondary to enzyme defect
239846006	Gout secondary to lead
239844009	Gout secondary to renal impairment
646651000000108	Gout Uncomplicated
48440001	Gouty arthritis
48440001	Gouty arthritis NOS
16015911000119102	Gouty arthritis of bilateral ankles
16015951000119101	Gouty arthritis of bilateral feet
16010751000119107	Gouty arthritis of bilateral great toes
16068491000119107	Gouty arthritis of bilateral knees
16015911000119102	Gouty arthritis of both ankles
16015951000119101	Gouty arthritis of both feet
16010751000119107	Gouty arthritis of both great toes
16068491000119107	Gouty arthritis of both knees
67148009	Gouty arthritis of great toe
1074021000119103	Gouty arthritis of left foot
1074031000119100	Gouty arthritis of left great toe
201670006	Gouty arthritis of multiple sites
48440001	Gouty arthritis of other specified site
1074001000119107	Gouty arthritis of right foot
1074011000119105	Gouty arthritis of right great toe
699681002	Gouty arthritis of temporomandibular joint
201669005	Gouty arthritis of the ankle and foot

201669005	Gouty arthritis of the ankle and/or foot
48440001	Gouty arthritis of the forearm
201666003	Gouty arthritis of the hand
48440001	Gouty arthritis of the lower leg
201667007	Gouty arthritis of the pelvic region and thigh
201663006	Gouty arthritis of the shoulder region
201663006	Gouty arthritis of the upper arm
428839004	Gouty arthritis of toe
190828008	Gouty arthropathy
239848007	Gouty bursitis
1073971000119100	Gouty bursitis of left olecranon
1073961000119106	Gouty bursitis of right olecranon
43193009	Gouty iritis
190829000	Gouty nephropathy
190829000	Gouty nephropathy NOS
190829000	Gouty nephropathy unspecified
9386003	Gouty neuritis
2740001	Gouty proteinuria
850991000006106	Gouty tophi + gout NOS
190842000	Gouty tophi of hand
27277001	Gouty tophi of heart
27277001	Gouty tophi of heart
1033961000000104	Gouty tophi of toe
3875003	Gouty tophus
402469004	Gouty tophus
16005991000119109	Gouty tophus of bilateral elbows
16076011000119100	Gouty tophus of bilateral feet
16006631000119109	Gouty tophus of bilateral hands
16005991000119109	Gouty tophus of both elbows
16076011000119100	Gouty tophus of both feet
16006631000119109	Gouty tophus of both hands

281362006	Gouty tophus of bursa
402470003	Gouty tophus of digit
190842000	Gouty tophus of hand
27277001	Gouty tophus of heart
16076051000119104	Gouty tophus of left elbow
16076171000119109	Gouty tophus of left foot
16006591000119109	Gouty tophus of left hand
281364007	Gouty tophus of olecranon bursa
14763005	Gouty tophus of pinna
281365008	Gouty tophus of prepatellar bursa
16076131000119106	Gouty tophus of right elbow
16075931000119103	Gouty tophus of right foot
16006551000119104	Gouty tophus of right hand
281363001	Gouty tophus of tendon
719680009	Gouty tophus of toe
24595009	Idiopathic gout
170731009	Initial gout assessment
283839008	Intercritical gout
284680007	Interval gout
170733007	Joints gout affected
284680007	Latent gout
239846006	Lead gout
239846006	Lead-induced gout
3875003	Other specified gouty manifestation
90560007	Other specified gouty manifestation NOS
46785007	Precocious adolescent gout
310101000119107	Primary chronic gout without tophus
309661000119108	Primary chronic gout without tophus of ankle and/or foot
309681000119104	Primary chronic gout without tophus of elbow
309701000119101	Primary chronic gout without tophus of hand
309721000119105	Primary chronic gout without tophus of hip

309741000119104	Primary chronic gout without tophus of knee
309901000119103	Primary chronic gout without tophus of multiple sites
310061000119109	Primary chronic gout without tophus of shoulder
310081000119100	Primary chronic gout without tophus of vertebra
310121000119103	Primary chronic gout without tophus of wrist
24595009	Primary gout
69896004	Rheumatic gout
239846006	Saturnine gout
710734008	Secondary chronic gout without tophus
239843003	Secondary gout
402469004	Tophus co-occurrent and due to gout
16005991000119109	Tophus of bilateral elbows co-occurrent and due to gout
16076011000119100	Tophus of bilateral feet co-occurrent and due to gout
16006631000119109	Tophus of bilateral hands co-occurrent and due to gout
281362006	Tophus of bursa co-occurrent and due to gout
402470003	Tophus of digit co-occurrent and due to gout
14763005	Tophus of ear co-occurrent and due to gout
190842000	Tophus of hand co-occurrent and due to gout
27277001	Tophus of heart co-occurrent and due to gout
16076051000119104	Tophus of left elbow co-occurrent and due to gout
16076171000119109	Tophus of left foot co-occurrent and due to gout
16006591000119109	Tophus of left hand co-occurrent and due to gout
281364007	Tophus of olecranon bursa co-occurrent and due to gout
281365008	Tophus of prepatellar bursa co-occurrent and due to gout
16076131000119106	Tophus of right elbow co-occurrent and due to gout
16075931000119103	Tophus of right foot co-occurrent and due to gout
16006551000119104	Tophus of right hand co-occurrent and due to gout
281363001	Tophus of tendon co-occurrent and due to gout
719680009	Tophus of toe co-occurrent and due to gout
3875003	Urate tophus
28428009	Visceral gout

48440001 [X]Gouty arthrpathy due/enzym defects+oth inherit disordr CE

Supplementary Data S2. Comorbidity definitions

Chronic kidney disease (CKD)

Definition - ever or current diagnostic code (see below) for CKD stages 3 to 5, renal failure, dialysis or a renal transplant at the time of index gout diagnosis, and/or two consecutive estimated glomerular filtration rates <60 ml/min/1.73 m^2 closest to the gout index diagnosis date (assuming they were within 5 years of diagnosis).

Hypertension

Definition - ever or current diagnostic code for hypertension at the time of index gout diagnosis. Absence of the comorbidity was assumed if a diagnostic code was not present.

Diabetes mellitus

Definition - ever or current diagnostic code for diabetes mellitus at the time of index gout diagnosis. Absence of the comorbidity was assumed if a diagnostic code was not present.

Ischaemic heart disease

Definition - ever or current diagnostic code for ischaemic heart disease at the time of index gout diagnosis. Absence of the comorbidity was assumed if a diagnostic code was not present.

Stroke or transient ischaemic attack

Definition - ever or current diagnostic code for a stroke or transient ischaemic attack at the time of index gout diagnosis. Absence of the comorbidity was assumed if a diagnostic code was not present.

Heart failure

Definition - ever or current diagnostic code for heart failure at the time of index gout diagnosis. Absence of the comorbidity was assumed if a diagnostic code was not present.

Obesity

Definition – recorded body mass index ≥30 kg/m2 on the reading closest to the index gout diagnosis date (assuming this reading was within 5 years before or after the diagnosis date).

Urolithiasis

Definition - ever or current diagnostic code for urolithiasis at the time of index gout diagnosis. Urolithiasis was assumed not present in the absence of a diagnostic code.

Alcohol excess

Definition - ever or current diagnostic code for alcohol excess or an alcohol-related problem at the time of index gout diagnosis. Absence of the comorbidity was assumed if a diagnostic code was not present.

Smoking status

Definition - ever or current diagnostic code for being an ex-smoker or current smoker at the time of index gout diagnosis.

Diuretic therapy

Definition - prescription issued for a diuretic medication (furosemide, bendroflumethiazide, spironolactone, bumetanide, indapamide, hydrochlorothiazide, eplerenone, metolazone, amiloride, torasemide, chlortalidone, benzthiazide or xipamide) within 4 months of the index gout diagnosis date.
